# Bison alter the northern Yellowstone ecosystem by breaking aspen saplings

**DOI:** 10.1002/ece3.10369

**Published:** 2023-08-29

**Authors:** Luke E. Painter, Robert L. Beschta, William J. Ripple

**Affiliations:** ^1^ Department of Fisheries, Wildlife, and Conservation Sciences Oregon State University Corvallis Oregon USA; ^2^ Department of Forest Ecosystems and Society Oregon State University Corvallis Oregon USA

**Keywords:** aspen, bison, conservation conflicts, ecological engineer, trophic cascade, Yellowstone National Park

## Abstract

The American bison (*Bison bison*) is a species that strongly interacts with its environment, yet the effects of this large herbivore on quaking aspen (*Populus tremuloides*) have received little study. We documented bison breaking the stems of aspen saplings (young aspen >2 m tall and ≤5 cm in diameter at breast height) and examined the extent of this effect in northern Yellowstone National Park (YNP). Low densities of Rocky Mountain elk (*Cervus canadensis*) after about 2004 created conditions conducive for new aspen recruitment in YNP's northern ungulate winter range (northern range). We sampled aspen saplings at local and landscape scales, using random sampling plots in 87 randomly selected aspen stands. Across the YNP northern range, we found that 18% of sapling stems had been broken. The causal attribution to bison was supported by multiple lines of evidence: (1) most broken saplings were in areas of high bison and low elk density; (2) saplings were broken in summer when elk were not foraging on them; (3) we directly observed bison breaking aspen saplings; and (4) mixed‐effects modeling showed a positive association between scat density of bison and the proportion of saplings broken. In a stand heavily used by bison, most aspen saplings had been broken, and portions of the stand were cleared of saplings that were present in previous sampling in 2012. Bison numbers increased more than fourfold between 2004 and 2015, and their ecosystem effects have similarly increased, limiting and in some places reversing the nascent aspen recovery. This situation is further complicated by political constraints that prevent bison from dispersing to areas outside the park. Thus, one important conservation goal, the preservation of bison, is affecting another long‐term conservation goal, the recovery of aspen and other deciduous woody species in northern Yellowstone.

## Graphical abstract

We documented American bison (*Bison bison*) breaking the stems of quaking aspen (*Populus tremuloides*) saplings (young aspen >2 m in height) and examined the extent of this nonconsumptive effect on aspen recruitment in northern Yellowstone National Park. As bison numbers in northern Yellowstone have greatly increased since the early 2000s, these large herbivores have begun to reduce aspen recruitment, limiting and in some places reversing a nascent aspen recovery.

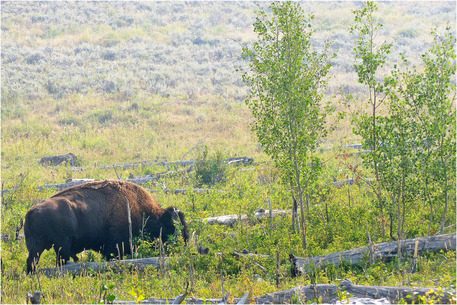

## 1 INTRODUCTION

The American bison (*Bison bison*), or American buffalo, has been called an ecosystem engineer and a keystone species because of the role of bison in shaping plant communities, and their interactions with other animal species (Boyce et al., 2022; Geremia et al., 2019; Gerlanc & Kaufman, 2003; Joern, 2005; Knapp et al., 1999; Krueger, 1986). For example, grazing and browsing combine with fire to stimulate productivity and increase the diversity of forbs and grasses, a dynamic observed in grassland ecosystems around the world (Knapp et al., 1999; Larson, 1940; McNaughton, 1984; Sinclair et al., 2010). Herbivory and fire also can increase habitat heterogeneity and prevent the invasion of grasslands by shrubs and trees. Research has often focused on bison in grassland ecosystems, their primary habitat. Less studied and understood are the effects of bison in woodland plant communities.

Deciduous woody plants typically comprise only a small portion of bison diet (Singer & Norland, 1994), and these plants may be rare in some grassland environments (Blackburn et al., 2020). The amount of browsing varies with season and availability of shrubs and other forage (Bork et al., 2013; Larter & Gates, 1991; Peden, 1976; Waggoner & Hinkes, 1986; Zeigenfuss & Schoenecker, 2021). These consumptive effects can be significant where bison are present in high densities year after year, as in northern Yellowstone (Beschta et al., 2020; Painter & Ripple, 2012; Rose & Cooper, 2016). Browsing often removes new shoots, and repeated browsing can suppress growth and prevent the recruitment of new trees.

Nonconsumptive physical effects of bison on woody plants including rubbing, horning, and trampling, add to the ways in which bison maintain and expand grasslands as ecological engineers (Reynolds et al., 2003). Bison can kill mature trees by rubbing and horning, thereby removing bark and girdling the trees (Coppedge & Shaw, 1997; Soper, 1941). Meagher (1973) found that “nearly every tree” had lost bark due to bison in extensive areas of lodgepole pine (*Pinus contorta*) forest in Hayden Valley, central Yellowstone National Park (YNP). In the same area, McHugh (1958) reported that 23% of trees with bark damage had been completely girdled and died or would likely die, while Olenicki and Irby (2005) found that 44% of pine saplings had been killed along the forest margin of the same area. In the Lamar Valley of northern YNP, Beschta et al. (2020) reported that 97% of mature lodgepole pine trees in their study area had been killed by bark removal. Bison begin rubbing trees in spring, as they are shedding winter hair, and continue this behavior into the summer. During the rut in late summer, bulls scrape bark from trees with their horns and break the branches of saplings and shrubs. Where bison are free to roam large areas, their dispersal can diffuse and reduce overall impacts (Soper, 1941), but where bison are confined to a smaller range or congregate in high densities, they can suppress or eliminate trees and shrubs.

In presettlement times, bison may have limited the extent of quaking aspen (*Populus tremuloides*) in the Aspen Parkland of the Canadian Northern Plains (Campbell et al., 1994). Bork et al. (2013) compared the effects on aspen of Rocky Mountain elk (*Cervus canadensis*) and bison where both ungulates were present. Herbivory was primarily attributed to elk, though bison also browsed. Physical damage and sapling mortality, including broken leader stems, were associated with bison. Their study took place in summer, and so did not consider differences in winter foraging behavior, when most aspen browsing occurs and elk strip and eat aspen bark (DeByle & Winokur, 1985).

**FIGURE 1:**
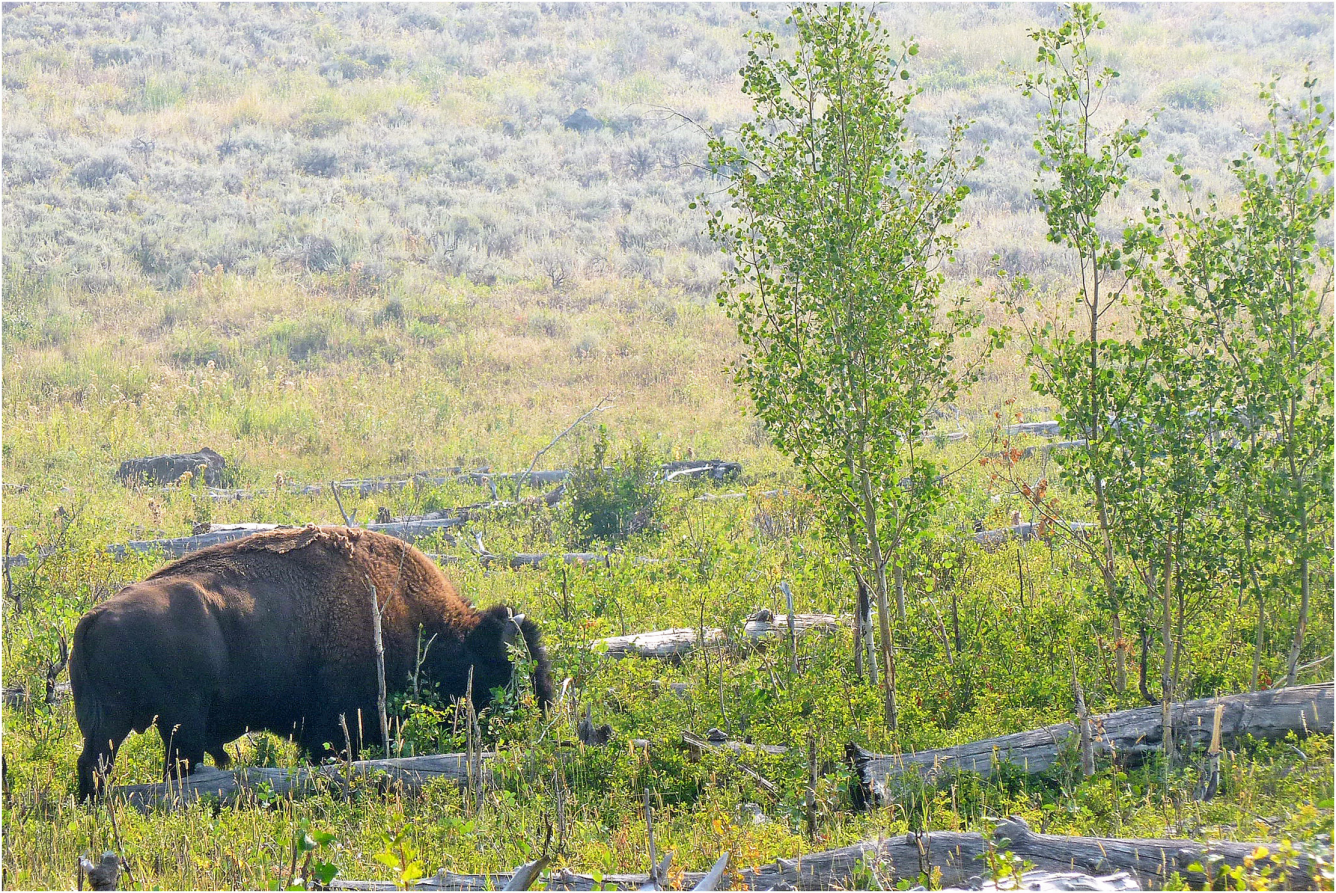
Bison bull browsing and breaking aspen saplings in the Lamar Valley in northern Yellowstone National Park, as shown in Video 1. Overstory aspen trees have died and fallen to the ground as seen in the photograph, and tall saplings have grown since the early 2000s. Broken stems of aspen saplings are visible in the foreground.

**FIGURE 2:**
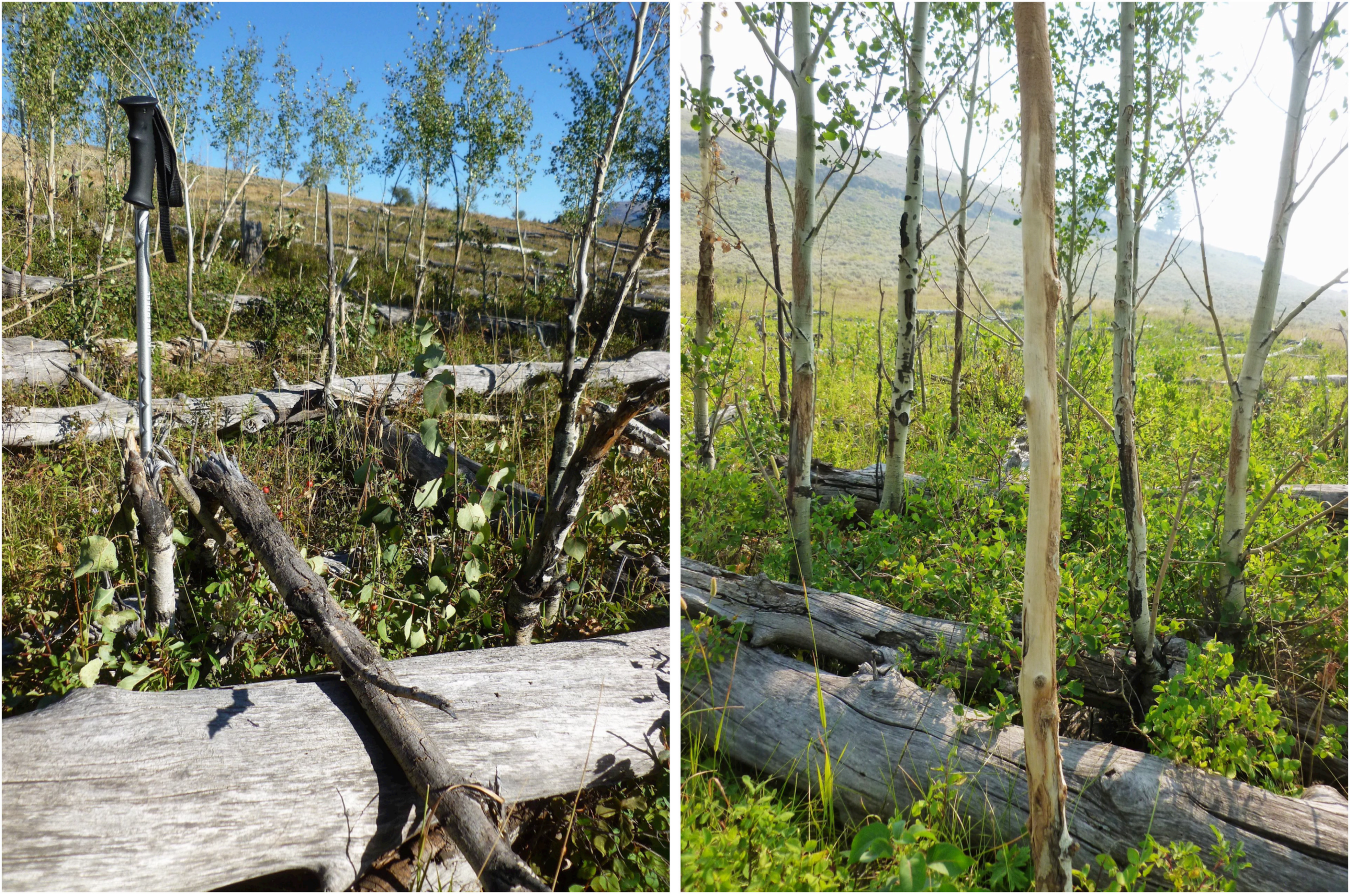
Live, broken, or stripped and girdled saplings in the Lamar East stand in September 2020. Fallen overstory aspen trees make access more difficult for elk and bison, providing partial protection to aspen saplings. We placed a sampling transect through the portion of the stand with the most saplings. Left: The walking stick marks two broken saplings about 4 cm in diameter, and another in the right foreground, in the Lamar East stand. Right: Some saplings were killed by girdling when the bark was scraped off, probably by bison horns. There were no tooth marks as would be left by elk, and the bark appeared to have been removed by abrasion (photos: L.E. Painter).

We documented a process by which bison killed aspen saplings (young aspen taller than 2 m and ≤5 cm in diameter at breast height), thereby reducing potential aspen recruitment. These saplings were tall enough to escape most browsing by elk and bison, but bison broke off the main stem at a low height (Figures 1 and 2). Others were stripped of bark, also killing the sapling. We recorded a video showing a bull bison breaking aspen saplings (Video 1) and assessed the local and large‐scale extent of this bison effect as part of a survey of northern range aspen stands. Was this breaking of saplings an interesting but overall insignificant behavior or was it potentially affecting aspen recruitment, demonstrating a process by which bison expand and maintain open grassland in a patchy woodland ecosystem?

**VIDEO 1:** In Yellowstone National Park's Lamar Valley, a bison bull breaks three tall aspen saplings in less than 4 min (video is edited to less than 2 min). Broken aspen stumps are visible in the foreground, as well as some aspen killed by horning that stripped off the bark. The bison in the video browsed on aspen branches and broke the main stem of the saplings by twisting or bending them over with mouth and horns. There were many branches at low height, so there was no need to break the saplings to browse aspen. The bison ate only a small portion, if any, of a broken sapling before moving on to another, thus, its behavior seemed focused on breaking saplings rather than accessing browse. We have seen only bulls breaking aspen saplings, not females. Video by Luke Painter, August 21, 2020.

### 1.1 Study area and aspen ecology

Quaking aspen is a clonal species that commonly reproduces by root sprouts (suckering), and stands of aspen are often a single organism connected by a common root system (DeByle & Winokur, 1985). Fire stimulates aspen reproduction from both roots and seeds, though browsing by elk and other large herbivores can suppress young plants (Smith et al., 2016; White et al., 1998). Aspen stands in northern Yellowstone have persisted through climate variations (Ripple & Larsen, 2000), and thus, they occur in places with adequate moisture to survive droughts. Recent trends toward a warmer and drier climate may cause additional stress (Kulakowski et al., 2013; Worrall et al., 2013), but in northern Yellowstone herbivory has been the primary factor limiting the growth of aspen from sprouts to saplings, and eventually trees (Beschta et al., 2016; Kay, 2018; Painter et al., 2014, 2018).

Aspen stand regeneration in northern YNP was suppressed for much of the 20th Century, primarily due to herbivory (browsing) by elk gathering during winter in the northern Yellowstone elk winter range, or “northern range” (Houston, 1982; Jonas, 1955; NRC, 2002; Wagner, 2006). Prior to this, more frequent fires and low herbivore densities likely favored aspen recruitment. In the early 1900s, elk numbers increased in the park following the removal of indigenous people, protection from other hunters, extirpation of gray wolves (*Canis lupus*), and reduction of other large predators. From the 1930s through the 1960s, park managers culled elk and bison to reduce browsing and grazing effects, but young aspen were still suppressed (Barmore, 2003). Culling in the park ended in 1968, and by the 1980s elk numbers had increased to record highs (Eberhardt et al., 2007; Wagner, 2006), eliminating almost all aspen recruitment by intensive browsing (Kay, 2001; Ripple & Larsen, 2000). By the late 1990s, many mature aspen trees were dying and most young aspen were less than 1 m tall, their summer growth removed by elk in winter (Larsen & Ripple, 2005). Thus, aspen stands on the northern range appeared to be dying out (NRC, 2002).

Wolves were reintroduced in the Yellowstone northern range in the mid‐1990s, adding to predation by bears, cougars, and human hunters outside the park, resulting in a significant reduction of elk density by the early 2000s (Eberhardt et al., 2007; White et al., 2012; White & Garrott, 2005), as well as changes in elk distribution and foraging behavior (Beschta et al., 2018; Kohl et al., 2019; Painter et al., 2015). Browsing pressure was reduced and more spatially variable, and subsequent aspen studies reported new recruitment of aspen saplings (Beschta et al., 2023; Painter et al., 2014, 2015; Ripple & Beschta, 2007, 2012). Bison, however, substantially increased in recent years (Mosley & Mundinger, 2018), as have their effects on aspen, particularly where bison congregate in summer as in the park's iconic Lamar Valley (Beschta et al., 2020; Kay, 2018).

**FIGURE 3:**
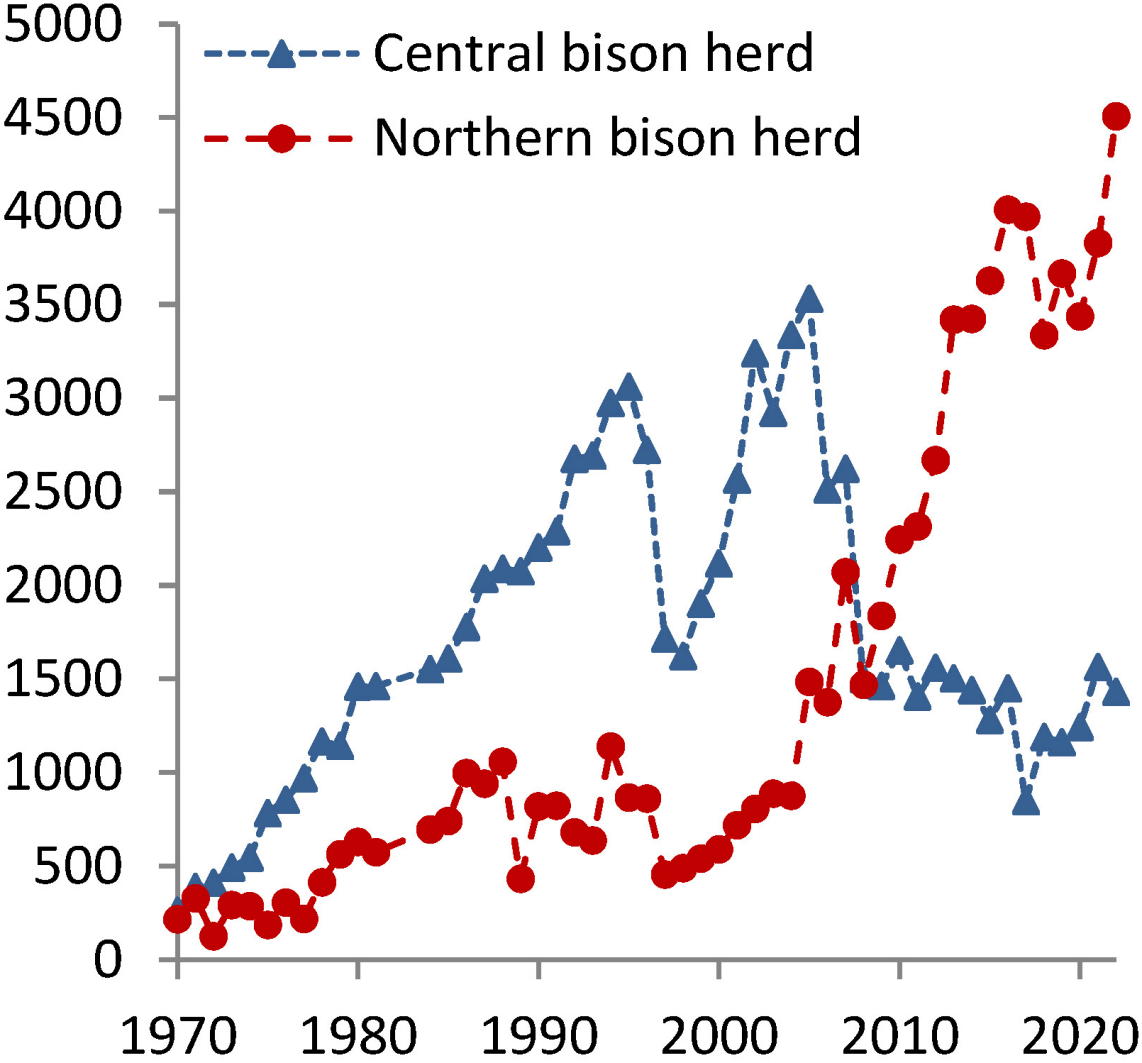
Annual maximum counts of bison in the central and northern regions of Yellowstone National Park, during June–August of 1970–2022 (Geremia, 2022). Our study area was in the range of the northern herd. Not shown are annual removals of bison; for example, more than 1000 bison were removed from the northern range in each year of 1997, 2006, 2008, 2017, and 2018.

After culling of bison and elk ended in 1968, bison increased much more slowly than elk. By the late 1980s, several hundred bison wintered in the northern range, and bison began gathering annually in Lamar Valley during the summer breeding season (Gates et al., 2005; Taper et al., 2000), forming a large herd that is now a well‐known tourist attraction. Bison on the northern range have increased from less than 1000 in the early 2000s to approximately 4000 animals in recent years (Figure 3) despite annual culling in winter as bison migrated outside the park boundary (Beschta et al., 2020; Geremia, 2022; Mosley & Mundinger, 2018; Plumb et al., 2009). By 2010, bison were the dominant large herbivores in much of the northern range.

## 2 METHODS

### 2.1 Data collection and analysis

Following methods of previous aspen recruitment surveys in the northern range, we defined saplings as young aspen >2 m tall and ≤5 cm in diameter at breast height (dbh; Kay, 1985, 1990; Larsen & Ripple, 2005; Painter et al., 2014). In the last decade since our 2012 sampling, some saplings have grown to be >5 cm dbh and we categorized these as young trees (6–20 cm dbh). “Overstory trees” refers to the older cohort of aspen trees that have been dying out, which were >20 cm dbh.

We collected data at two different spatial scales: a single focal stand in an area of high bison use, and random sampling of stands across the northern range. The focal stand was the location of a long‐term aspen monitoring transect established in 1958 (Barmore, 2003), which we called the Barmore Transect. We called this stand Lamar East, after the Lamar East exclosure, to which it was compared by Barmore. When we sampled the Lamar East stand in September 2020, it had many saplings and was frequented by herds of bison throughout the summer. We recorded the heights and browse status of all aspen in the original Barmore Transect. In addition, we placed a belt transect, 4 m × 60 m, through the portion of the stand with the greatest density of saplings (Figure 2). For each live sapling in the transect, we recorded dbh in cm at 1.4 m height, the standard height for measuring tree diameter, and also diameter at 0.5 m height (*d*_0.5_, cm), measurable on most broken sapling stumps. We used the relationship between these measurements to estimate the dbh of broken saplings based on their diameter at 0.5 m height. We recorded *d*_0.5_ for each broken young aspen >2 cm. We measured the dbh of saplings girdled, but not broken. From these data, we calculated the percentage and size range of saplings whose stems had been broken or killed by girdling in this stand.

**FIGURE 4:**
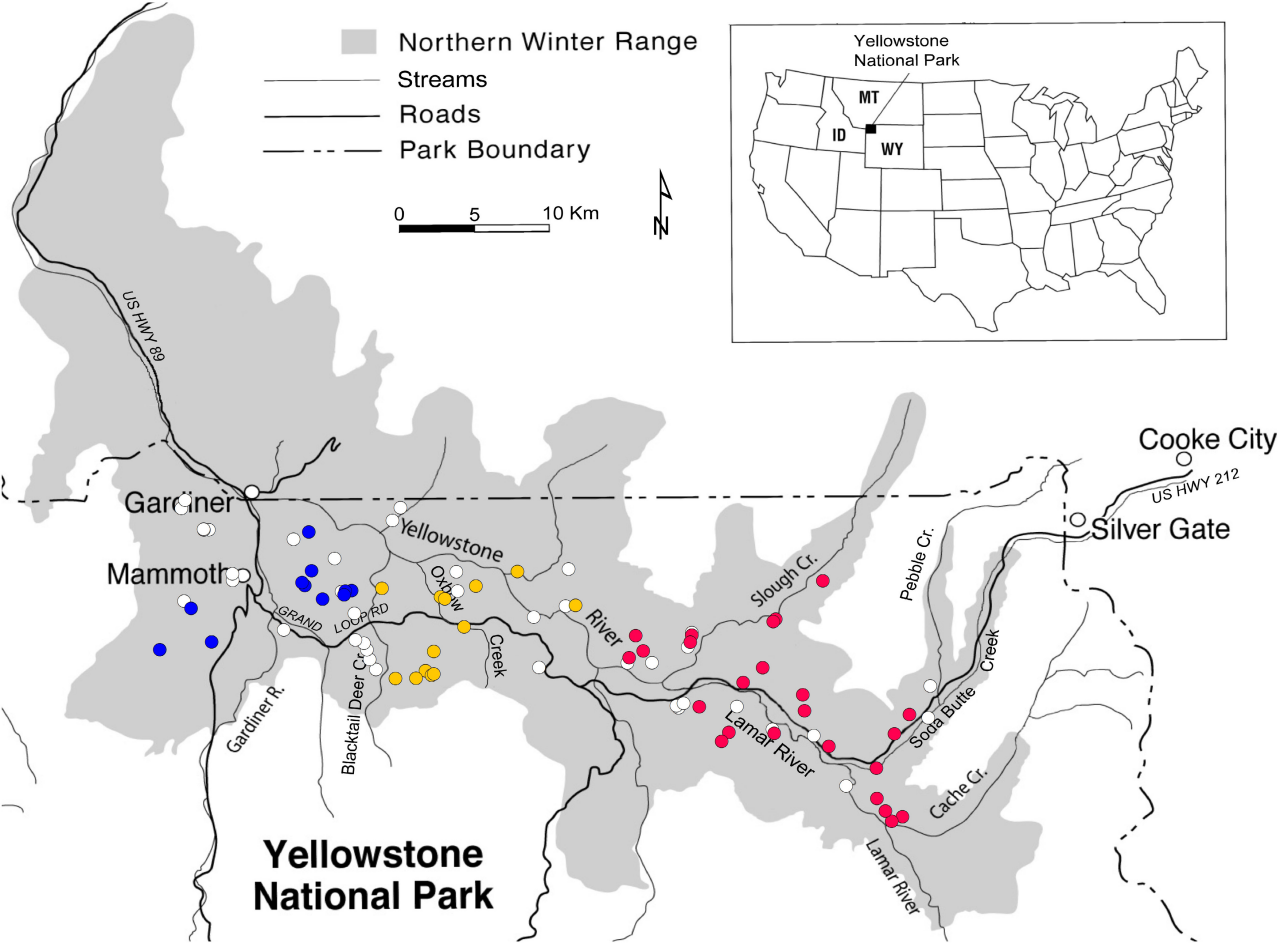
Map of locations where random sampling plots contained saplings (*n* = 48 of 87 plots). Colors indicate groupings by sector: West (blue), Central (yellow), and East (red). Sector was used as a random effect in mixed‐effects modeling. White circles indicate sampling locations where the plots did not contain saplings.

At a landscape scale, in late August and early September of 2020 and 2021 we monitored aspen sapling recruitment in 87 aspen stands (Figure 4), randomly selected and previously surveyed in 1997–1998 by Larsen and Ripple (2005) and in 2012 by Painter et al. (2014). We relocated stands using GPS coordinates from 2012, and in each placed a sampling plot 2 m wide beginning at the live, dead, or fallen aspen tree nearest the GPS location. This plot extended 30 m toward the centroid of the stand. In the plot, we counted the number of live saplings, broken saplings (>2 cm *d*_0.5_), and saplings killed by bark girdling. We indexed fecal pile density (regardless of age) of bison and elk using two 2 × 50 m transects, spaced 10 m apart, parallel to and approximately 10 m from the edge of each stand, in xeric grassland or forest edge. Mesic sites or wet meadows were avoided for scat transects, due to the low likelihood of scats being detected in those conditions. Fecal piles were identified to species (genus for deer, *Odocoileus* spp.) based on pellet and pile morphology (Elbroch, 2003).

We assessed the extent of ungulate‐caused mortality of aspen saplings as the total percentage of saplings that had been killed by breaking or bark removal, out of all saplings live or dead. A broken sapling was considered dead as a sapling even if it resprouted from the base. The standard error for sample proportion *p* was calculated as (*p*(1−*p*)/*n*)^0.5^, multiplied by a *z*‐factor of 1.96 to estimate 95% confidence intervals (CI).

As in previous northern range studies, we divided the study area into three sectors for comparison: West, Central, and East (Painter et al., 2015; White et al., 2012). The East sector was where elk densities decreased most rapidly and to the lowest levels in the early 2000s and where new aspen saplings first appeared in response (Painter et al., 2015; Ripple & Beschta, 2007). However, the East sector also was where bison increased the most in recent years and where we observed the greatest mortality of aspen saplings in preliminary surveys.

Antler rubbing by elk occurs in late summer, whereas our study area had few elk in summer, particularly in the East and Central sectors. Thus, scraped and rubbed aspen saplings were more likely to be from bison. Broken saplings were attributed to bison for four reasons:

1. Broken saplings were found in areas frequented by bison, or where there was evidence of bison activity (scat, tracks, and sightings). If elk were the cause, there should have been more broken aspen in the West sector where elk were more common, but the opposite was the case.
2. Bison were present in large numbers on the northern range in summer (Painter & Ripple, 2012), and this was when many of the aspen saplings were broken, as evidenced by attached foliage. In contrast, elk are known to use the northern range primarily in winter, when they browse and strip aspen as other forage is unavailable (Barmore, 2003; Houston, 1982; Painter et al., 2015).
3. Bison are known to break tree saplings (Bork et al., 1997; Coppedge & Shaw, 1997), but this is not a behavior reported for elk (Keigley & Frisina, 2008).
4. We directly observed bison breaking saplings, as in Video 1. We previously observed bison breaking down tall willow bushes (*Salix* spp.) (Painter & Tercek, 2020).

We tested for an effect of elk or bison scat density (fecal piles/100 m^2^) on the proportion of aspen saplings broken, using logistic mixed‐effects modeling for binomial distribution, with R Statistical Software (version 4.2.3) package lme4 (Bates et al., 2015; R Core Team, 2023). We included sector (West, East, and Central) as a random effects variable (Figure 4), to account for differences in the effects of bison or elk not explained by scat density and varying by sector. For example, herds of bison composed mostly of females and calves were more common in the East and Central sectors, but we have never observed females or calves breaking aspen, only bulls. In contrast, bulls wander individually over the whole study area. Thus, there could be a component of scat variation by sector that does not affect the breakage of aspen, accounted for by the random effects variable.

## 3 RESULTS

### 3.1 Lamar East focal stand

**FIGURE 5:**
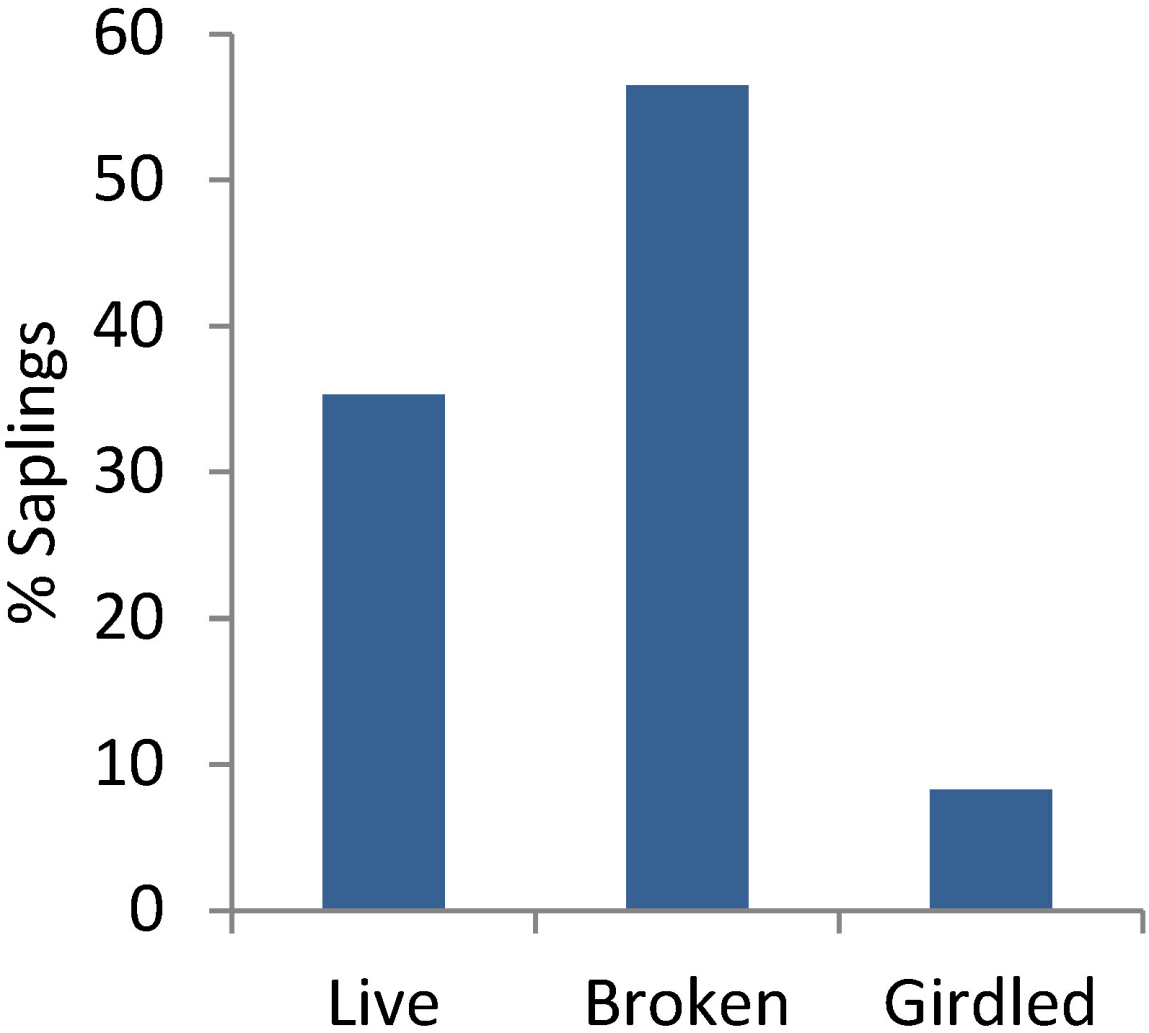
Percentages of aspen saplings live, broken, or girdled (dead from the stripping of bark) in a 4 × 60 m belt transect in the Lamar East stand (*n* = 83). This stand was in the East sector of the northern range, an area used by herds of bison throughout the summer. Although aspen saplings in this stand emerged after browsing by elk was greatly reduced in the early 2000s, bison recently have killed most of them (see Video 1).

In 2020, the sapling transect in the Lamar East stand included 83 saplings (live, dead, or broken; Table 1) of which 58% (*n* = 48) had been broken. An additional 8% (*n* = 7) had been stripped and girdled, for a total of 66% of saplings that had been killed (Figure 5). The remaining live saplings (*n* = 28) ranged from 0.8 to 5.3 cm dbh, plus two young trees (>5.5 cm). The estimated mean dbh of broken saplings was 2.3 cm (range 1.2–4.6), using the relationship: dbh = 0.88*d*_0.5_–0.53; *n* = 30, *r* ^2^ = .90. We found no broken saplings, in this stand or elsewhere, with actual or estimated dbh larger than 5 cm. However, larger young trees would still be vulnerable to bark stripping.

**TABLE 1:** Three sampling transects in the Lamar East stand monitored in 2020.

Transect	Year	Plot size	Trees	Young trees	Live saplings	Broken saplings	Stripped saplings	All saplings
Barmore Transect	1958, 1965	6 × 100 ft	4, 2	0	0	0	0	0
Barmore Transect	2020	6 × 100 ft	0	0	0	0	0	0
Random plot	1998	2 × 30 m	13	0	0	0	0	0
Random plot	2012	2 × 30 m	0	0	6	0?	0?	6
Random plot	2020	2 × 30 m	0	0	0	4	0	4
Sapling transect	2020	4 × 60 m	0	2	28	48	7	83

*Note*: The Lamar East stand was the subject of two long‐term studies, and in 2020, we added a third transect in the area of greatest sapling density. The Barmore Transect results are shown for 1958 and 1965 (Barmore, 2003); we relocated and sampled these transect markers in 2020. Larsen and Ripple (2005) placed a random plot in the Lamar East stand in 1998 that we relocated in 2012 (Painter et al., 2014) and 2020 by GPS. Columns list the name of the belt transect, the year it was monitored, dimensions of the plot, and number of aspen overstory trees (>20 cm dbh, estimated from photos); young trees (6–20 cm dbh); live saplings (>2 m height and ≤5 cm dbh); broken saplings at least 2 cm diameter; dead saplings with stripped and girdled bark; and all saplings, the sum of these three sapling categories. Dead saplings were not recorded in the 2012 survey, but none were noted in site photos or descriptions.

**FIGURE 6:**
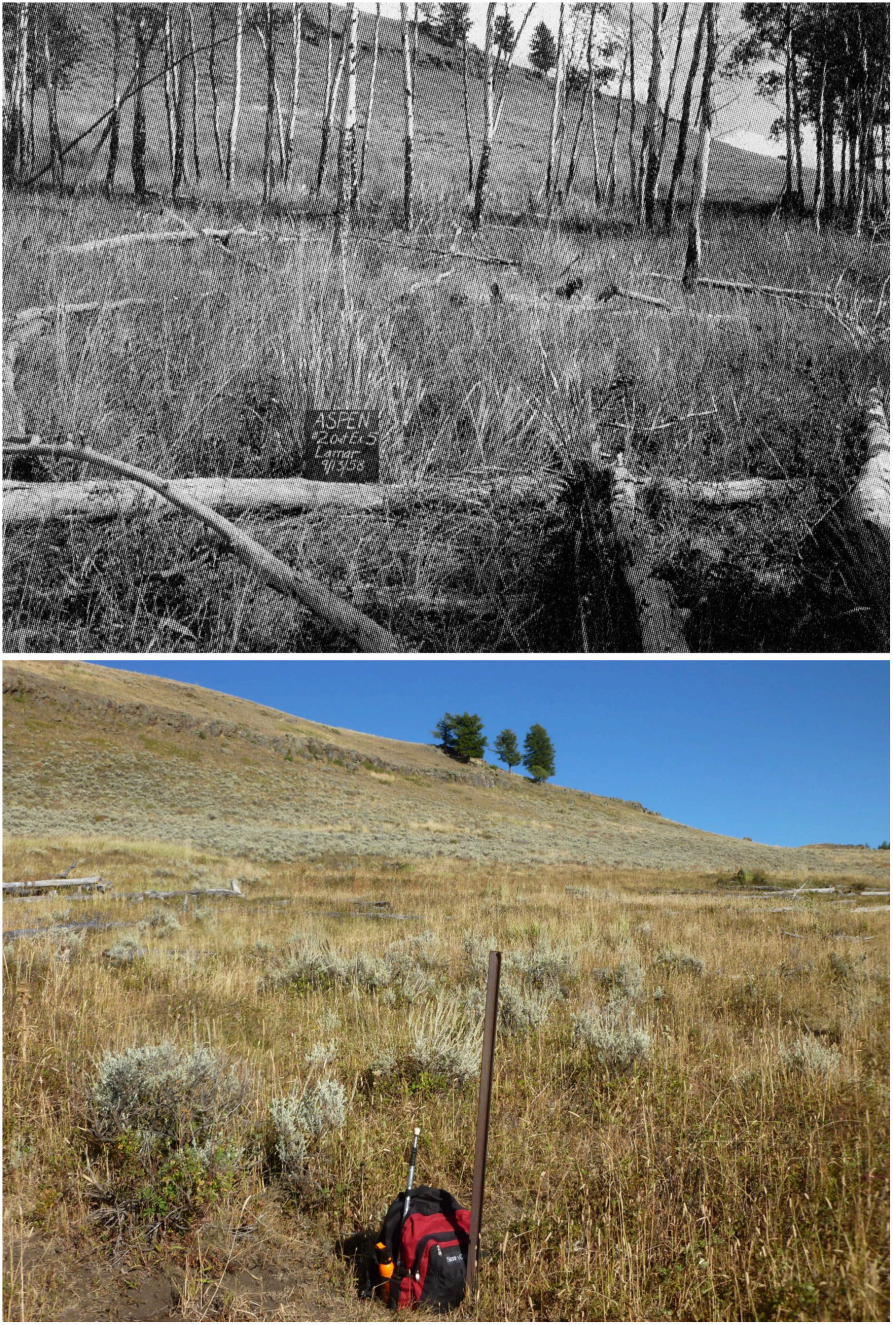
Photographs of the Barmore Transect in 1958 (top) and 2020 (bottom), in the Lamar East aspen stand (Barmore, 2003). Most overstory trees had died and fallen many years before 2020, as little remained of them on the ground. Trees were not replaced by new recruitment due to browsing that suppressed the growth of young aspen sprouts, formerly by elk and more recently by bison. The metal marker stake was at GPS location: north 4969772, east 562759, wgs84 zone 12T; rebar stakes marking the other end of the 1.8 × 30.5 m (6 × 100 ft) transect were found due east of the first marker (photos: L.E. Painter).

We relocated the Barmore Transect using historical photographs (Figure 6) and found the metal marker stakes. The 1958 photograph shows that the transect went through a group of overstory trees that are now dead. Barmore reported that sprouts in 1958 and 1965 were heavily browsed and averaged <0.5 m tall. We found this still to be the case in 2020, but in 1958 and 1965 aspen sprouts numbered about 60 in the transect. In 2020, the transect was on the edge of the stand and contained no live trees or saplings (Table 1, Figure 6), and only seven aspen sprouts (five of which were new summer growth) all <0.5 m tall; five of the total, or 71%, had been browsed in the summer of 2020.

Our random, long‐term monitoring plot in the Lamar East stand was surveyed in 1998, 2012, and 2020 (Table 1). In 1998, it contained no aspen saplings and all sprouts were <1 m tall, but in 2012 the plot contained six saplings and 14 other young aspen taller than 1 m that might soon become saplings. In 2020, this plot contained four broken but no live saplings, clear evidence of a reversal in aspen sapling recruitment. This 10‐year comparison suggests that our 2020 sampling under‐represented the number of saplings that grew and then were lost, the small stumps having rotted away in the intervening period of time.

### 3.2 Landscape scale

**FIGURE 7:**
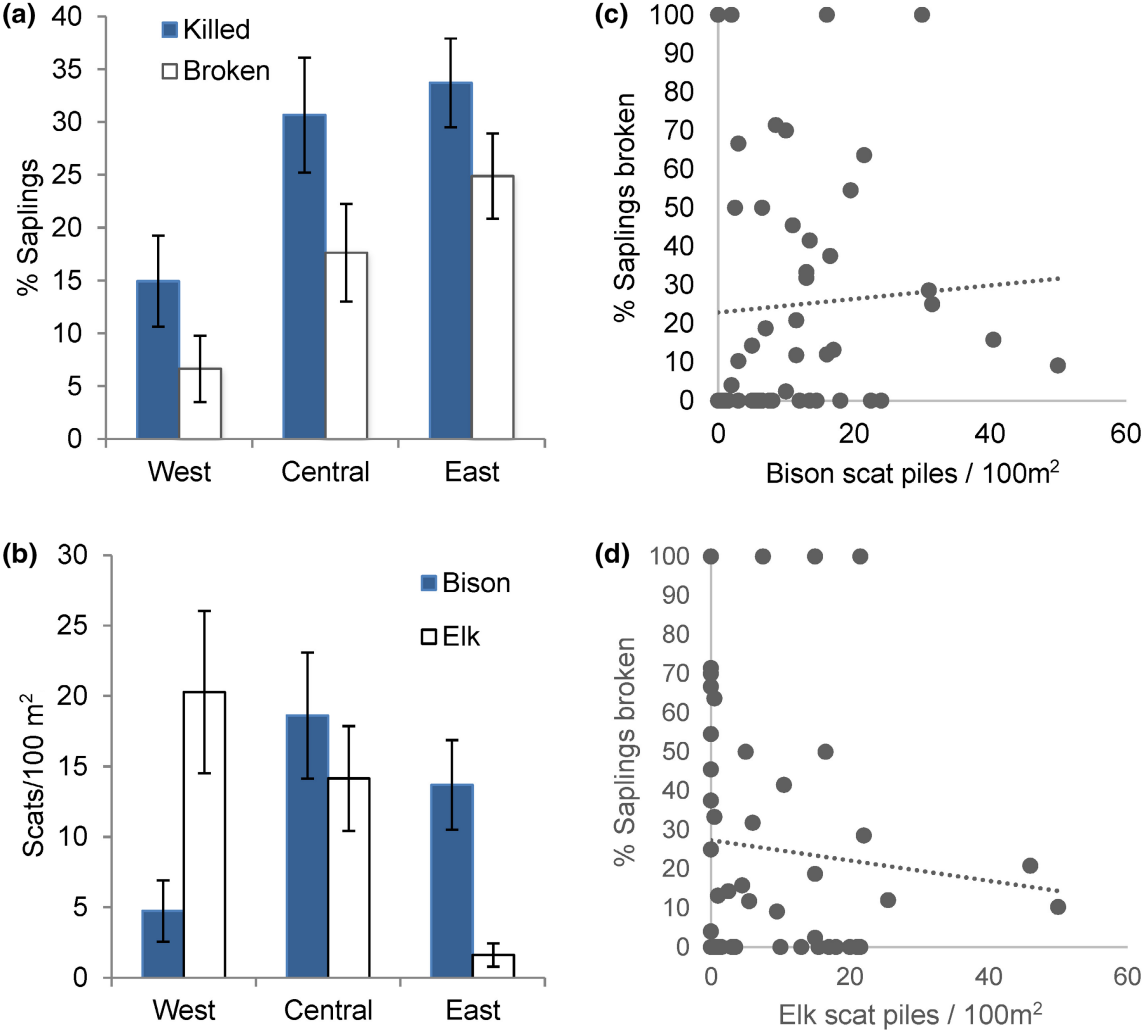
Results from random plots in 87 aspen stands in northern YNP. Error bars show 95% confidence intervals. West, Central, and East are the three sectors of the YNP northern range. (a) Blue bars show total percentage of saplings killed by either breakage or bark stripping (girdling). White bars show only those broken. Of 87 random sampling plots, 48 contained saplings, live, dead, or broken, while an additional 39 plots had no saplings (total saplings: West *n* = 241, Central *n* = 261, and East *n* = 442). (b) Scat density (fecal piles/100 m^2^) of bison or elk, counted in plots associated with the 87 sampled aspen stands. (c) Scatter plot of percentage of saplings broken as a function of bison scat density, with trendline. (d) Scatter plot of percentage of saplings broken as a function of elk scat density.

At a landscape scale, our random sampling plots in 2020 included 679 live saplings and 265 dead, totaling 944 saplings in 48 of the 87 plots. The remaining 39 plots had no saplings, live or dead. Broken saplings comprised 18% (*n* = 172 of 944), while those dead with stripped bark were 10% (*n* = 93 of 944). Percentages killed and broken by range sector are shown in Figure 7a and the scat density for elk and bison in Figure 7b. We occasionally detected scat piles of pronghorn (*Antilocapra americana*), moose (*Alces alces*), or deer (*Odocoileus* spp.), but these were not included in the totals due to their very low numbers.

Preliminary analysis suggested a weak relationship between fecal pile density and the proportion of saplings broken (Figure 7c,d). Mixed‐effects modeling showed a significant effect (>95% confidence) on the proportion of broken saplings from scat density of bison but not elk (Table 2).

**TABLE 2:** Results of logistic mixed‐effects modeling.

Proportion broken	Estimate	*p*‐value	Variance	SD
Intercept	−2.01319	<.001		
Bison fecal piles/100 m^2^	0.02905	.03		
Elk fecal piles/100 m^2^	−0.00351	.79		
Sector (intercept)			0.277	0.4558

*Note*: The response variable was the proportion of aspen saplings broken out of all saplings (live, broken, or stripped) in random plots, in randomly selected aspen stands. After removing plots that had no saplings, live or dead, 48 plots remained. Column 1 lists the two independent fixed‐effects variables, which were the fecal pile densities of bison and elk, and also the random effects variable sector (West, Central, and East). Column 2 lists estimates for the intercept and the beta coefficients for the two independent variables. Column 3 lists *p*‐values for statistical significance, and Columns 4 and 5 the variance and standard deviation of sector as a random effect (*n* = 3).

## 4 DISCUSSION

### 4.1 Causes of sapling mortality

On the YNP northern range, aspen saplings were rare during much of the 20th century due to intensive browsing by elk, but this began to change as elk densities decreased inside the park following wolf reintroduction in the late 1990s as part of large carnivore restoration (Painter et al., 2015; Ripple & Beschta, 2007). Our results suggest that many saplings grown since that time have been broken down by bison, and others were killed by bison and elk removing bark. Although broken saplings may resprout from the base, the tall sapling has been lost and new sprouts are vulnerable to browsing and trampling. We found no evidence of significant sapling mortality from other causes (e.g., disease or drought) in our monitored stands, but we observed two other stands where saplings had been cut by beaver.

Elk do not normally break aspen saplings, but elk may strip the bark to eat in winter, girdling and killing the saplings. Large numbers of stripped saplings would be expected where winter elk densities had recently increased (Keigley & Frisina, 2008), as we observed in Dome Mountain Wildlife Management Area north of the park (Painter et al., 2018). However, in northern YNP the number of elk wintering in the park actually decreased recently to historically low numbers, particularly in the East sector (Painter et al., 2015, 2018; White et al., 2012), where we saw the greatest mortality of aspen saplings (Figure 7a). Many saplings were broken and most of this damage occurred in areas of low elk density, facts that point to bison as the agent responsible (Bork et al., 2013). Moose may break aspen saplings to access browse in winter (Telfer & Cairns, 1978), but moose numbers have been very low in our study area (Mosley & Mundinger, 2018), and many saplings were broken in summer with little browsing. In contrast, bison have greatly increased in recent years (Figure 3) and were frequently observed in or near aspen stands in summer. Bison scat and tracks were evident in our sites, and we directly observed bison breaking aspen saplings (Figure 1, Video 1). Multiple lines of evidence support the conclusion that bison were responsible for most if not all of the broken saplings and many with girdled bark as well.

The bison bull seen in Video 1 ate only a small portion of a broken sapling, if any, before moving on to another. Therefore, the purpose of the behavior seemed to be to break the saplings rather than to access browse. We observed only bulls engaging in this behavior, so it may be related to displays of aggression. Similarly, male elephants may break down trees but feed little on them, suggesting a social or sexual reason for this behavior (Midgley et al., 2005).

### 4.2 Effect on aspen recruitment

The proportion of saplings broken, and therefore the effect on aspen recruitment, varied greatly by location. Bison have substantially reduced potential recruitment in some northern range aspen stands, particularly in the Lamar Valley. For example, Video 1 shows a field of broken sapling stumps, suggesting that saplings once covered much more of the stand area, and a transect through the remaining patch of saplings showed that 58% had been broken (Table 1, Figures 1 and 2). These results suggest that bison are likely to prevent the stand from replacing its dead overstory trees. In Video 1, a bison bull broke down three aspen saplings in less than 4 min. Multiplied over months and years, and hundreds of bison, this process could have a significant effect on aspen recruitment in that vicinity. This raises an interesting question: If bison are now removing aspen saplings faster than they can grow in some stands, how did saplings grow there? What can explain the growth of aspen saplings after decades of absence and then their loss? We suggest that three important trends converged to favor aspen recruitment in the decade 2000–2010, particularly in the East sector of the YNP northern range:

1. Lower densities and changes in behavior of wintering elk following large carnivore restoration (Kohl et al., 2018; Painter et al., 2015, 2018; White et al., 2012).
2. Relatively low bison numbers, before the dramatic increase in recent years (Figure 3).
3. Large numbers of dying aspen trees falling to the ground (Beschta & Ripple, 2020; Larsen & Ripple, 2005) limiting stand access. These fallen trees provided temporary protection for young aspen from bison and elk (Beschta et al., 2020; Ripple & Larsen, 2001), as elk densities decreased.

This “sweet spot” in time for aspen recruitment resulted in new saplings arising in many stands, particularly where large numbers of mature trees fell to the ground (Figure 2). Subsequently, two additional changes resulted in the loss of many of the recently grown saplings: (1) As fallen trees decayed, aspen saplings became more accessible, and (2) concurrently bison became more numerous (Figure 3).

The role of fallen trees as protection for young aspen has been noted in previous studies. Ripple and Beschta (2007) and Painter et al. (2015) found that heights of young aspen were positively correlated with the presence of fallen trees. Also, placement of large woody debris is a technique commonly used to discourage browsing and aid aspen recruitment (Kota & Bartos, 2010). With the high elk densities of the 1980s and 1990s in northern Yellowstone, starving elk consumed nearly all available aspen sprouts, but as elk numbers decreased and foraging behavior changed during the early 2000s, browsing rates decreased to moderate levels of about 40%–60% of sprouts browsed annually (Painter et al., 2015). In this situation, even small, localized reductions in browsing intensity, such as those provided by fallen trees, can facilitate the growth of young aspen (Beschta et al., 2018, 2020; Kuijper et al., 2015; Ripple & Larsen, 2001). Thus, fallen trees can play an important role in aspen recruitment, an opportunity that is lost after the overstory trees in a stand are gone.

Our random sampling across the northern range provided an assessment of the broader extent of nonconsumptive effects of bison on aspen saplings. In the East sector, where bison were common and elk few, about 25% of all saplings found were broken, and an additional 9% killed by bark stripping (Figure 7). Furthermore, our long‐term data suggest that these percentages are underestimated. We found similar sapling mortality in the Central sector, which may reflect the recent expansion of the northern range bison population (Figure 3). This contrasts with the West sector, where elk were much more common as shown by annual counts (Painter et al., 2015) and our scat transects, and bison less common, and only about 7% of saplings were broken. Thus, the effects of bison on aspen saplings appear to be significant and expanding westward from the area of greatest bison density in the Lamar Valley.

From the 1930s to the present day, concerns about the suppression of aspen, willow, and cottonwood (*Populus* spp.) on the northern range have focused on the elk that wintered there (Houston, 1982; NRC, 2002; Painter et al., 2018; Wagner, 2006). Only recently, as elk have decreased and bison increased, have bison emerged as an important factor affecting the dynamics of these woody plants (Beschta et al., 2020; Painter & Ripple, 2012; Ripple et al., 2010; Rose & Cooper, 2016). Their impact on aspen saplings adds a new complication to the assessment of potential aspen recruitment. In some places, the additional mortality may be within the range of usual thinning by stem competition, but in some stands in the East sector, such as our focal Lamar East stand, nearly all saplings in large portions of a stand had been broken. Further research into the thinning process as aspen mature could help to assess the ecological significance of these effects. Unlike stem competition, bison effects are often greatest on the edges of dense sapling patches, limiting the area covered by saplings and eventually trees. In addition to the northern range, bison may also be an important influence in the Lamar River headwaters (e.g., Mirror Plateau) where for decades they have gathered in summer breeding herds (Gates et al., 2005; Meagher, 1973; Taper et al., 2000).

### 4.3 Bison past, present, and future in Yellowstone

Bison numbers inside the park were likely relatively low in prehistoric and presettlement times, based on paleological and historical evidence, although herds were common in the lower‐elevation valleys outside the park (Beschta & Ripple, 2019; Keigley, 2019; Meagher, 1973; Mosley & Mundinger, 2018; Schullery & Whittlesey, 2006). Northern YNP is relatively high in elevation with extreme winter conditions compared to bison ranges on the plains. Another factor likely to have kept prehistoric bison numbers low in the park area was hunting by indigenous people who resided there or traveled through the Yellowstone northern range to hunt bison in southwestern Montana (Nabokov & Loendorf, 2004). Through most of the history of the park in modern times, elk were the dominant large herbivores. In the early 2000s, as elk browsing decreased, relatively low bison numbers (Figure 3) appear to have been more compatible with the growth of young aspen and willow than the high numbers of the recent decade. Furthermore, Yellowstone's bison have not been allowed to migrate outside the park as their density increases. By agreement with the State of Montana, bison have been confined to the park vicinity (Gates et al., 2010; Plumb et al., 2009; White et al., 2015), and hundreds are culled annually when they leave the park. Thus, bison are not free to disperse like other wildlife species, a fact that may have implications for the ecology of the park.

The management plan for Yellowstone bison has been under review to develop new goals and policies and assess environmental impacts (YNP, 2022). Alternatives under consideration would maintain the current number of about 4000–5000 bison or increase this target number. One option would allow bison to increase to a food‐limited carrying capacity estimated at more than 8000 bison, nearly twice the recent number. There is no proposed option to reduce bison densities. Thus, the likely outcome of this plan will be expanding and intensified impacts on aspen and other woody plants in northern Yellowstone. Public notice documents for the new plan indicate that “potential impacts of bison grazing” would be expected, but these are unspecified. The substantial effects of bison on the long‐term park goal of recovering aspen and willow on the northern range (NRC, 2002) appear to be left out of this important discussion that will determine the future of bison in the Yellowstone ecosystem.

## 5 CONCLUSION

Bison maintain and expand grasslands by suppressing and removing woody vegetation. They do this by browsing young shoots of woody species, breaking branches and stems, and girdling trees by horning and rubbing. These effects can be significant in places where bison have a strong and continuous presence, as in the Lamar Valley of northern Yellowstone. In contrast, the ecological effects of prehistoric bison on the western plains were diffused over large areas by seasonal migrations (McHugh, 1972), a fact that can make modern localized bison herds, confined by physical or political boundaries, different from the free‐ranging bison of the past (Kohl et al., 2013; Wuerthner, 1998). The conservation of bison in Yellowstone is an important success story, as is the recovery of aspen and other deciduous woody plants that began after restoration of the park's large carnivores. Researchers are only beginning to understand how these conservation goals have overlapped and affected each other. As efforts mount to restore bison to more of their former habitat (Freese et al., 2007), awareness of the many ways bison shape their environment is important to guide expectations for and management of these iconic animals.

## AUTHOR CONTRIBUTIONS

**Luke E. Painter:** Conceptualization (lead); data curation (lead); formal analysis (lead); investigation (lead); methodology (lead); resources (equal); writing – original draft (lead); writing – review and editing (lead). **Robert L. Beschta:** Conceptualization (equal); funding acquisition (lead); methodology (equal); project administration (equal); resources (equal); writing – review and editing (supporting). **William J. Ripple:** Conceptualization (supporting); methodology (supporting); project administration (equal); writing – review and editing (supporting).

## Data Availability

Supporting data are archived in the ScholarsArchive@OSU, Oregon State University: Painter, L.E.; R.L. Beschta; W.J.R. Ripple. 2023. Northern Yellowstone aspen sapling data collected 2020–2021. Oregon State University Scholars Archive. http://doi.org/10.7267/r207tx72t.
